# Exercise Intervention Improves the Metabolic Profile and Body Composition of Southwestern American Indian Adolescents

**DOI:** 10.15436/2376-0494.16.1180

**Published:** 2016-11-02

**Authors:** Leslie Colip, Mark R. Burge, Phillip Sandy, Donica Ghahate, Jeanette Bobelu, Thomas Faber, Vallabh Shah

**Affiliations:** 1 School of Medicine, University of New Mexico Health Sciences Center, Albuquerque, NM; 2 Indian Health Services Comprehensive Centre in Zuni Pueblo, Zuni, New Mexico

**Keywords:** Pediatric, Obesity, Prediabetes, Fitness, Native American

## Abstract

**Introduction/Purpose:**

The Southwestern American Indian population carries a high prevalence of metabolic syndrome and obesity, placing this group at higher risk than the general population for developing early type 2 diabetes and cardiovascular disease, likely impacting overall lifespan. This study aims to evaluate the impact of early lifestyle interventions which promote healthy eating and regular exercise on risk factors contributing to the development of the metabolic syndrome among the adolescent Zuni Pueblo population.

**Materials and Methods:**

We describe a prospective, single site, community-based cohort study performed among sixty-five adolescent Zuni Indians aged 13.9 ± 1.7 years who were recruited between March 2011 and January 2014. The study intervention consisted of a targeted, tri-weekly exercise regimen with nutritional counselling, and the primary study outcomes included changes from baseline in metabolic profile (fasting lipids, A1c), vital signs (blood pressure, resting heart rate) and anthropometric characteristics of the study group.

**Results:**

41 participants have anthropometric data measured at baseline and after completion, biochemical data are available from 30 participants, and body composition data from 26 patients. Using the paired Student’s t-test with Bonferroni correction, significant improvements were shown in pediatric BMI percentile, fasting lipid profile, A1C, total body fat, and fat free mass after six months of exercise and nutritional intervention.

**Conclusions:**

A simple, standardized fitness program among Southwest American Indian adolescents was effective at reducing fasting lipids and adiposity, as well as improving glycemic indices over the course of six months.

## Introduction

Obesity is a serious and growing health concern among American Indian youth. An increased prevalence of risk factors associated with cardiovascular disease and chronic diseases such as Type 2 Diabetes and metabolic syndrome has occurred in this population over recent years. The 2010 - 2011 NHANES (National Health and Nutrition Examination Survey)study found that 34.5% of US children aged 12 - 19 years met criteria for being overweight or obese (BMI > 85^th^ percentile), and the prevalence of obesity is higher among low-income families and in the American Indian population ^[1–3]^. Recent epidemiological data specific to the adolescent Southwest American Indian population since the 1990s are lacking, but the Navajo Health and Nutrition Survey (1991 - 1992) showed that among 160 adolescents aged 12 – 19, 35% of the boys and 40% of the girls were over-weight (BMI > 85^th^ percentile for NHANES II)^[4]^. The increased prevalence of obesity over the past few generations is generally attributed to increased access to high calorie food, sugar sweetened beverages and an increase in sedentary lifestyles^[5]^.

The Zuni Indians reside in a small pueblo located in rural New Mexico, with more than 90% of all Zunis living within the boundaries of the pueblo^[6]^. This tribal community faces economic disadvantages as well as a history of healthcare disparities. As a result, the Zuni population is experiencing epidemic rates of obesity, diabetes, and the associated health consequences of these diseases^[6]^. Nationwide, obese children are shown to have an increased relative risk of mortality and death from cardiovascular disease after reaching adulthood, particularly in men above the age of 45^[7, 8]^. Efforts to curb this growing problem focus on the critical need for lifestyle modification, including exercise and dietary changes early in life. Many small studies have investigated the effect of exercise and nutrition intervention in subsets of this population, but systematic reviews of these trials fail to identify a clearly efficacious treatment approach due to small sample sizes^[9]^. The largest study was performed among 1,704 elementary school aged Navajo children via the Pathways Program^[10]^. The intervention in this program focused on dietary changes, increased physical activity while at school, a healthy lifestyle classroom curriculum, and the involvement of the participant’s family. Unfortunately, after 3 years of study, participants did not show any improvement in the prevalence of obesity or any increase in physical activity.

We postulate that an after school program with a supervised exercise regimen and nutrition counselling will result in decreased BMI and obesity rates, as well as reduced metabolic risk factors for diabetes and early cardiovascular disease, among Zuni adolescents.

## Materials and Methods

This study was approved by the University of New Mexico Health Sciences Center Human Research Review Committee and the Indian Health Service Institutional Review Board, and all participating children rendered written informed assent and consent. Study participants received $25 each time they participated.

### Exercise and nutrition intervention

Sixty-five adolescent subjects were recruited from one middle school and one high school in Zuni Pueblo, New Mexico. The inclusion age range was 12 – 17 years of age. Participants were recruited though school health classes, distribution of flyers, home visits or by the invitation of a friend. The participating population was invited to participate in an after school exercise program at the Zuni Health Initiative’s Wellness center, a community based recreational center funded by an NIH grant through the Department of Health and Human Services. Participants were transported after school three times a week to participate in the program which consisted of sixty-minute exercise sessions including a ten-minute warm-up and three 15-minute rotating activities that incorporated aerobic exercise (jogging, stationary bike, treadmill or elliptical, and/or calisthenics), resistance training and group exercise (group games). These sessions were led and supervised by the study-affiliated Community Health Representatives (CHRs) who live in the Zuni community. Participants also attended a monthly diet and nutritional education session led by a registered dietician from the Indian Health Services. Additionally, parents attended an instructional session on healthy eating and preparing nutritional sack lunches for their children.

### Metabolic profile

All participating children were assessed at baseline, 12 weeks and 24 weeks with fasting blood work to assess changes in metabolic risk factors, including a fasting lipid panel, A1C, liver function tests, uric acid, and urinary albumin to creatinine ratio. Samples were obtained *via* venipuncture by a certified community health representative who then processed the samples and sent them for clinical chemistry measurements performed at the CLIA (Clinical Laboratory Improvement Amendments) certified Tricore Reference Laboratory (Albuquerque, NM) using standard clinical assays.

### Anthropometric parameters

Participants underwent vital sign determination (heart rate, blood pressure, height and weight) in addition to a body composition analysis by direct measurement of waist and arm circumference, as well as by bioelectrical impedance (Quantum II, RJL Systems, Clinton Township, MI) at baseline, 12 and 24 weeks. Participants’ body-mass index (BMI) and BMI-percentile were calculated using the CDC Children’s BMI Tool for Schools. In the pediatric population, children are defined as being “at risk for overweight” when body-mass index was between the 85^th^ and 95^th^ percentiles. The term “overweight” is applied to children with a BMI in excess of the 95^th^ percentile for children of the same age and sex^[9, 11, 12]^. BMIs in excess of the 99^th^ percentile are associated with “severe obesity.”11 Normal weight is defined as BMI between the 5^th^ and 85^th^ percentiles^[9, 11, 12]^.

Previous studies have shown that BMI is a poor estimator of body fat and obesity status in this population, but multi-frequency Bioelectrical Impedance Analysis (BIA) has been validated in the pediatric American Indian population as a minimally invasive technique to analyze body composition according to total body fat, fat free mass, and body water percentages^[10, 13, 14]^. This measurement accurately assesses different populations using population-specific means, as opposed to methods such as waist circumference or pediatric BMI assessment. Although increases in visceral fat enhance the likelihood of morbidity in the adult population, no widely accepted clinical measure of central adiposity yet exists for children. Waist and arm circumference were also collected at all three time points.

#### Statistical analysis

Comparisons between baseline, 12 and 24 weeks for all members of the cohort were performed using the paired Student’s t-test with Bonferroni correction for the primary outcomes. Due to the high dropout rate, an analysis comparing baseline values between the cohort dropouts and those participants who completed 24 weeks (denoted as “Final Cohort”) was performed using the unpaired Student’s t-test in order to assess for differences in baseline characteristics between those groups. The 12-week Cohort and the Final Cohort were defined as the most complete data sets at each time point. Additionally, an intention to treat analysis was performed using the last observation carried forward (LOCF) technique and compared with the baseline characteristics of the whole cohort using the paired Students t-test. Analysis was performed using SAS Version 9.4 (Cary, NC). Statistical significance was assessed using Bonferroni correction (α/2) and was defined as p < 0.025. Results are reported as Mean ± SD throughout the text, tables and figures.

## Results

Mean age for the whole cohort was 13.9 ± 1.7 years and participants were predominantly male (62.12%). Metabolic data, vital signs, and anthropometric and BIA data at baseline, 12, and 24 weeks for the Whole Cohort are shown in Tables 1 and 2. These tables also include the results of the intention to treat analysis at 24 weeks. Final data across the cohort were obtained from between 29 and 41 participants, as shown in Table 3. The variation in sample size is attributable to partial data collection amongst participants due to either refusal of the participant to undergo venipuncture or failure to participate in other assessments (e.g. body measurements, BIA analysis). As depicted in Tables 1 and 2, the 12-week cohort incorporated 40 subjects and the Final Cohort included data from 30 participants. Due the low final population size, the baseline characteristics of the whole cohort did not accurately reflect the characteristics of the participants who actually completed the study, veiling certain improvements. As such, an *ad hoc* analysis of the Final Cohort at baseline and 24 weeks is shown in Table 3. A comparison between participants who left the study and those who remained in the study is also shown in Table 3. This analysis demonstrated that participants who dropped out of the study had lower a BMI and more desirable HDL and triglyceride concentrations than those who completed the study.

### Anthropometric Measures and Body Composition

**Figure 1:**
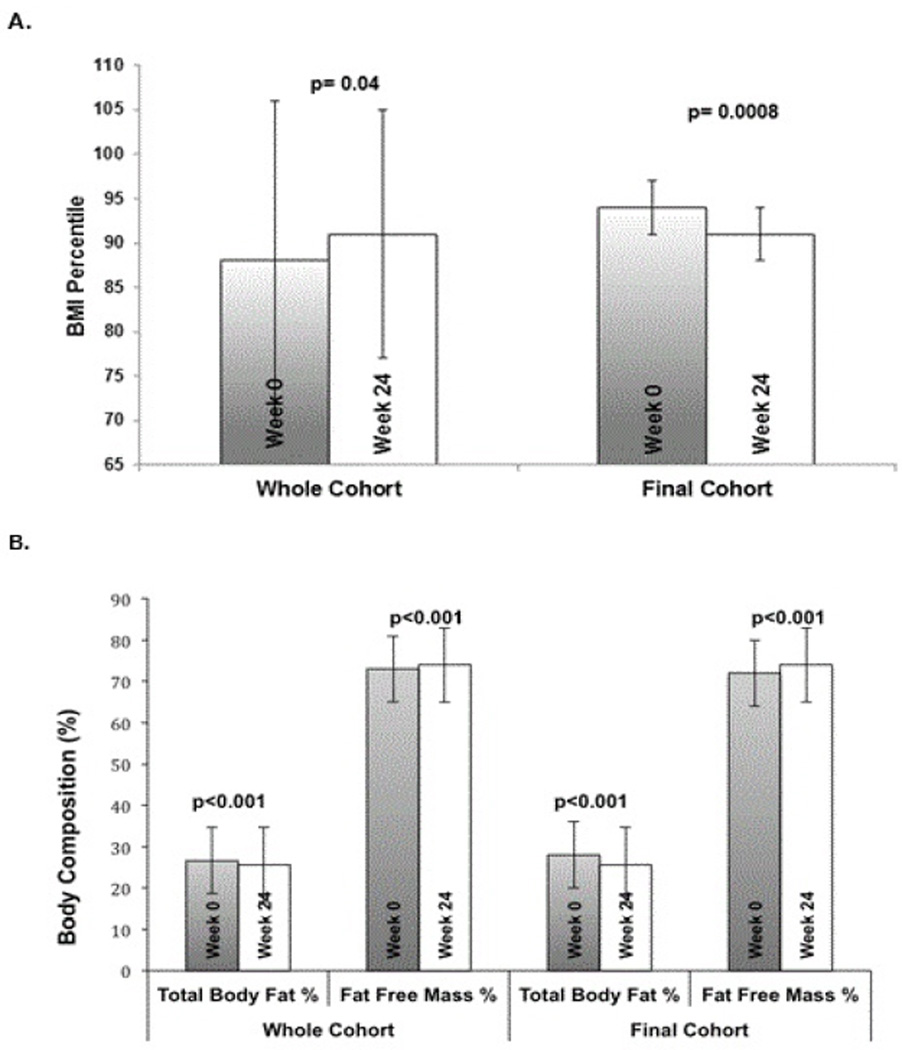
Summary of Pediatric BMI percentile (1A) and body composition as measured by BIA (1B) in the entire cohort versus the Final Cohort after 24-weeks. Comparison is between the entire cohort (including dropouts) versus the Final Cohort who completed the 24-week program.

The mean pediatric BMI percentile for the whole cohort showed an overall increase over the course of the study, but this was found to be due to the lower body weights and BMI percentiles in the group of participants who dropped out of the study (Table 2). Post hoc analysis of the Final Cohort showed significant improvements in weight and pediatric BMI. After the 24-week intervention, weight decreased from 81.2 ± 18.1 to 78.0 ± 12.2 kg (a 4% decrease, p < 0.0001). The intention to treat analysis also showed significant improvements (Table 1). Although the Final cohort still had a mean BMI percentile in the obese range after 24-weeks, there were significant improvements from the 94^th^ to the 91^st^ percentile (a 3%-ile decrease, p < 0.0001). Figure 1A shows reductions in BMI percentile at 24 weeks compared to baseline for both the Whole Cohort and the Final Cohort. Changes in waist and arm circumference were not significant in any of the cohorts. Vital signs, including systolic, diastolic, mean arterial pressure and heart rate also did not show any significant change (Tables 1 and 3).

Body composition as measured by bioelectrical impedance showed significant improvements after the 24-week intervention, including a 2% reduction in total body fat percentage and a 2% increase in fat free mass percentage in the final cohort (p < 0.001; Figure 1B). Findings in the ITT cohort were similar.

#### Glycemic control

**Figure 2:**
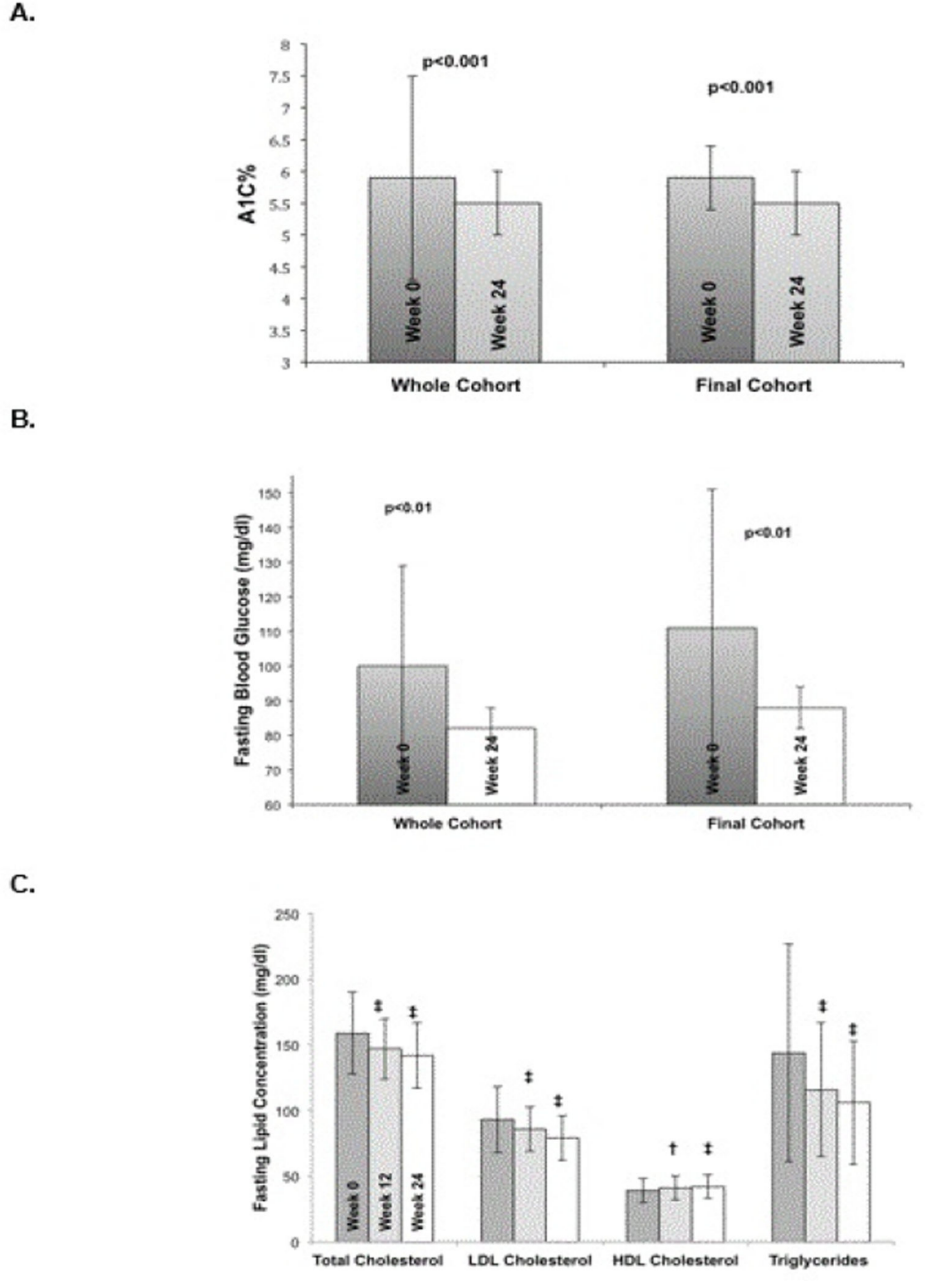
Summary of A1c (2A) and fasting blood glucose (2B) at baseline and after 24-weeks in the entire cohort versus the Final Cohort. (2C) Summary of fasting lipid panel results at baseline, 12 and 24 weeks for the Final Cohort. Comparison is between the entire cohort (including dropouts) versus the Final Cohort who completed the 24-week program (2A and 2B). Statistically significant changes (2C) between week 0 (dark gray) to 12 (light gray) and week 0 to 24 (white), with *= p < 0.025, † = p < 0.01, and ‡ = p < 0.001.

During the 24-week exercise and nutritional intervention, progressive and statistically significant improvements were seen in glycemic control as exhibited by improvements in mean A1c from 5.9 ± 2.0% at baseline (in the pre-diabetes range) to a mean of 5.5 ± 0.5% at 24 weeks (in the normal range; p < 0.001). The intention to treat analysis also showed a significant improvement to 5.7 ± 1.9% (p < 0.001). The Final Cohort showed similar results, with a baseline A1c of 5.9 ± 0.7% decreasing to 5.5 ± 0.5% at 24 weeks (p < 0.001). Fasting blood glucose levels improved significantly from a mean of 100 ± 29 mg/dl at baseline (in the impaired fasting glucose range) to a mean of 82 ± 6 mg/dl at 24 weeks (in the normal range; p = 0.003). The intention to treat analysis also showed improvement in fasting blood glucose to 89 ± 6mg/dl after 12 weeks (p < 0.001). The Final Cohort exhibited similar results with a baseline fasting blood glucose of 111 ± 40 mg/dl decreasing to 89 ± 6 mg/dl at 24 weeks (p < 0.01) as shown in Table 4, Figures 2A and Figure 2B.

#### Fasting lipids

Participants in all cohort sex hibited significant improvements in all lipid panel parameters (shown in Table 1 and Figure 2C), including an average 11% decrease in total cholesterol in the whole cohort, and a 13% decrease in the final cohort (163 ± 26 mg/dl at baseline vs.142 ± 25 mg/dl at 24 weeks, p < 0.0001). There was also an average 18% decrease in LDL cholesterol in the final cohort (from 96.3 ± 17 at baseline to 79 ± 17 mg/dl at 24 weeks, p < 0.0001). HDL cholesterol increased in the whole cohort from 39 ± 9 mg/dl at baseline to 42 ± 9 mg/ dl at 24 weeks (p < 0.0001), and an average 17% increase in the final cohort (from 36 ± 8 mg/dl at baseline to 42 ± 9 mg/dl at 24 weeks, p < 0.0001). Finally, there was a 26% decrease in triglyceride concentrations in the whole cohort (144 ± 83 mg/dl at baseline to 106 ± 47 mg/dl at 24 weeks, p < 0.0001) and a 38% decrease in the Final Cohort (171 ± 90 mg/dl at baseline to 106 ± 47 mg/dl at 24 weeks, p < 0.0001). Results were also significant in the ITT cohort, as shown in Table 1.

There was also a significant improvement in uric acid, a marker of systemic inflammation, in both the whole cohort (a 13% decrease, p < 0.01) and the ITT cohort (a 13% decrease, p < 0.01), as shown in Table 1. There was no statistical improvement in urinary albumin to creatinine ratio (UACR), creatinine, gamma-glutamyl-transferase (GGT) or serum albumin concentrations.

Due to the high dropout rate, baseline characteristics were compared between subjects who dropped out of the study and those who completed the study, the Final Cohort, as shown in Table 3. In general, the dropout population was found to be healthier than the Final Cohort, with a lower pediatric BMI: 78^th^ ± 24 percentile versus 94^th^ ± 3 percentile (p < 0.001). The was also a lower mean body weight in the dropout group: 68.0 ± 23.6 kg vs. 81.2 ± 15.4 kg (p = 0.04), and waist circumference was significantly less in the dropout group: 35 ± 7 inches vs. 41 ± 6 inches (p < 0.025). In terms of glycemic control, there was no difference between the dropout group and the Final Cohort with respect to A1c (5.6 ± 0.5% vs. 5.9 ± 0.7%, p > 0.05), but fasting blood glucose was significantly lower in the dropout group (90 ± 6 mg/dl vs. 111 ± 40 mg/dl, p < 0.01). The fasting lipid panel was more desirable in the dropout group as compared to the Final Cohort across all parameters, but only HDL cholesterol and triglyceride concentrations showed statistically significant differences, as shown in Table 3.

## Discussion

Adolescent obesity continues to be a difficult problem to address, particularly in high-risk populations such as the American Indian population, where obesity prevalence is estimated to be higher than the rest of the United States population^[1]^. Current recommendations for the treatment of adolescent obesity include 60 - 90 minutes of physical fitness daily, which can be difficult to achieve in any adolescent population, but particularly in health-disparate populations where resources are often limited.

Previous studies, such as the KLAKS study out of Germany, showed improvements in body composition, cardio-metabolic and glycemic control parameters after one year of exercise and lifestyle modification curriculum^[15]^. But these results have not proven to be directly translatable to the American Indian population, where the largest scale program to date, Pathways, failed to demonstrate a significant decrease in the prevalence of adolescent obesity^[10]^. The aim of this study was to demonstrate that an afterschool program consisting of tri-weekly, supervised, 60-minute exercise sessions (and adjunctive nutritional counselling) would have a beneficial effect on the anthropometric and cardio-metabolic parameters of adolescents from the Zuni Pueblo.

Our results are in contrast to the Pathways study in that our population had a 4% body weight reduction and a decrease in pediatric BMI percentile, from 94^th^ to 91^st^ percentile, among the participants who completed the study. BIA body composition analysis confirmed a significant decrease in total body fat percentage and an increase in fat free mass percentage. These differences may be due to variation in the interventions and/or the longer follow up period in the Pathways study; 3 years versus 6 months. Additionally, the current study’s intervention consisted of supervised and dedicated fitness sessions outside of school, which may lend itself to a more focused and rigorous exercise experience than a less supervised exercise intervention.

A more recent study analyzed 5,532 non-diabetic Pima Indian patients, aged 5 - 19 years, who were assessed for risk factors leading to the development of diabetes mellitus over a mean follow up 12.4 years^[16–18]^. In the 1,281 children who developed diabetes during the course of the study, BMI and glucose intolerance were closely associated with the development of diabetes. This study also demonstrated that children who developed metabolic abnormalities at a younger age had a higher risk of developing diabetes. Hyperlipidemia and hypertension were not correlated with an increased risk of the development of diabetes in this study.

The current study demonstrates pronounced and significant improvements in glycemic parameters as measured by A1c and fasting blood glucose concentrations. In the cohort of patients who completed the 24-week study, A1c and fasting serum blood glucose were, on average, normalized from the pre-diabetes and impaired glucose tolerance ranges, respectively, to the non-diabetic range. Total cholesterol, LDL cholesterol, HDL cholesterol and serum triglycerides all showed significant improvements. Even though this study does not contain any information about clinical outcomes, it seems likely that these findings will translate into lower rates of progression to type 2 diabetes, obesity, and early cardiovascular disease if they are sustained ^[19–21]^.

Limitations of this study include the lack of a control group and the high rate of non-completion. Nevertheless, one of the major concerns with a high dropout rate is worry that the effects of randomization will become invalidated, but since there was no randomization in the current study, this particular concern is not *apropos*. Additionally, longer follow up will be important to gauge whether or not the metabolic and anthropomorphic gains observed in this study are sustained. The high dropout rate in this study is possibly due to participants withdrawing due to scholastic reasons or due to graduation from school. The participants who dropped out of the study were shown to be, on average, a healthier group with respect to body composition and cardio-metabolic parameters than the Final Cohort, so it may be argued that a higher risk population was ultimately included in the Final Cohort of this study. Finally, there is also limited information on available on the value-added by the nutritional intervention towards the improvements observed in this study, if any^[22]^.

## Conclusion

The significant metabolic and body composition improvements demonstrated in this study argue convincingly that an after school exercise and nutritional intervention program offers the opportunity for improvement in some of the most important indicators of health among high risk American Indian adolescents, especially if such interventions are ultimately shown to reduce the morbidity and mortality associated with obesity and diabetes in this population.

### Future directions

We will focus on identifying suitable physical activities that are culturally appropriate and enhance adherence, as well as advocating for sustained funding for meaningful physical education training in the Zuni schools.

## Figures and Tables

**Table 1:** Baseline characteristics of the entire cohort versus weeks 12 and 24, and the Intention to Treat analysis.

	Whole Cohort			ITT
	Week 0	Week 12	Week 24	Week 24
**Sample Size**	65	40	30	65
**Age (years)**	13.9 (1.7)			13.9 (1.7)
**Male Sex (%)**	61.5%	60.00%	60.50%	61.50%
**Vitals**				
**MAP (mmHg)**	80 (9)	82 (8)	79 (7)	81(8)
**Mean SBP (mmHg)**	104 (20)	99 (34)	106 (9)	108 (12)
**Mean DBP (mmHg)**	66 (12)	68 (8)	66 (7)	67 (8)
**Pulse (BPM)**	76(5)	78 (6)	74 (6)	38(5)
**Body Composition**				
**Height (inches)**	62 (3)	62 (3)	63 (3)‡	62.7(3.2)‡
**Weight (kg)**	76.7 (24.5)	78.0 (22.7)‡	78.0 (24.0)‡	73.9 (23.6)‡
**Pediatric BMI %-ile**	88 (18)	92 (12)†	91 (14)	86.5 (19)‡
**Waist circumference**				
**(inches)**	39 (7)	40 (6)	40 (6)	39 (7)
**Glycemia**				
**A1c %**	5.9 (2)	5.6 (0.5)‡	5.5 (0.5)‡	5.7 (1.9)‡
**Fasting Blood Sugar**	100(29)	90 (8)†	82 (6)†	89 (6)†
**Lipids**				
**Total Cholesterol (mg/dl)**	159 (31)	147 (23)‡	142 (25)‡	147 (30)‡
**LDL Cholesterol (mg/dl)**	93 (26)	86 (17)‡	79 (17)‡	83 (24)‡
**HDL Cholesterol (mg/dl)**	39 (9)	41 (9)†	42 (9)‡	42(10)‡
**Triglycerides (mg/dl)**	144 (83)	116 (51)‡	106 (47)‡	109 (57)‡
**Other**				
**Cr (mg/dl)**	0.67 (0.14)	0.67 (0.13)	0.63 (0.17)	0.63 (0.17)
**UACR**	31 (44)	25 (42)	24 (38)	24 (38)
**Albumin (mg/dl)**	3.9 (0.2)	4.0 (0.3)	4.0 (0.3)	4.0 (0.3)
**Uric Acid (mg/dl)**	5.4 (1.3)	4.8 (1.3)*	4.7 (1.4)†	4.7 (1.4)†
**GGT (mg/dl)**	36(37)	33 (38)	37 (25)	37 (25)

Baseline subject who completed the 24-week study are designated as the Final Cohort. The intention to treat analysis depicts the results of the cohort at 24 weeks with LOCF as compared to baseline using the paired Student’s t-test with Bonferroni adjustment for multiple comparisons, with

* <0.025,

† p<0.01, and

‡ p<0.001. with

* p<0.025,

† p<0.01, and

‡ p<0.001.

**Table 2:** Changes in body composition in the whole cohort, and the Intention To Treat analysis.

	Whole Cohort			ITT
	Week 0	Week 12	Week 24	Week 24
**Phase Angle**	6.46 (0.77)	6.59 (0.84)‡	6.84 (0.99)†	6.76 (0.86)‡
**Total Body Fat %**	27.6 (8.4)	26.3 (8.0)‡	25.7 (9)‡	25 (9)‡
**Fat Free Mass %**	73.3 (8.5)	73.8 (8.1)	74.4 (9)‡	75 (9)‡

Statistically significant changes between weeks 0 to 12 and weeks 0 to 24 employing Bonferroni correction, with

* p<0.025,

† p<0.01, and

‡ p<0.001.

**Table 3:** Baseline characteristics of the Final Cohort versus subjects who dropped out prior to completion of the 24 week study week.

	Week 0			
	Final Cohort		Study Dropouts	
	n		n	
**Male Gender**	31	62.50%	34	61.70%
**Age (years)**	31	14 (2)	34	14 (2)
**Height (inches)**	41	62 (3)	23	62 (4)
**Weight (kg)**	41	81.2 (24.5)	23	68.0 (24.0)**
**Waist Circumference (in)**	41	40 (6)	21	35 (7)†
**Pediatric BMI %-ile**	41	94 (3)	23	78 (24)‡
**A1c %**	30	5.9 (0.7)	34	5.6 (0.5)
**Fasting Blood Sugar (mg/dl)**	30	111 (40)	33	90 (6)†
**Total Body Fat (%)**	26	28 (8)	32	25 (9)
**Fat Free Mass (%)**	26	72 (8)	32	74 (9)
**Phase Angle**	26	6.3 (0.9)	32	6.5 (0.7)
**Total Cholesterol (mg/dl)**	30	163 (26)	33	154 (34)
**LDL Cholesterol (mg/dl)**	29	96 (17)	34	90 (30)
**HDL Cholesterol (mg/dl**	30	36 (8)	33	42 (10)*
**Triglycerides (mg/dl)**	30	172 (90)	33	119 (67)*

Dropout cohort defined as participants who did not complete the 24-week study. Values listed as mean (±SD). Statistically significant changes between weeks 0 to 12 and weeks 0 to 24 were defined using Bonferroni correction with

* p<0.025,

† p<0.01, and

‡ p<0.001.

**Table 4:** Change in the Final Cohort; subjects who completed the 24-week program as compared to baseline.

	Final Cohort		
	Week 0		Week 24
	n		
**Height (inches)**	41	62 (3)	63 (3)‡
**Weight (kg)**	41	81.2 (24.5)	78.0 (12.2)‡
**Waist Circumference (in)**	41	40 (6)	40 (5)
**Pediatric BMI %-ile**	41	94 (3)	91 (3)‡
**A1c %**	30	5.9 (0.7)	5.5 (0.5)‡
**Fasting Blood Glucose (mg/dl)**	30	111 (40)	89 (6)†
**Total Body Fat (%)**	26	28 (8)	26 (9)‡
**Fat Free Mass (%)**	26	72 (8)	74 (9)‡
**Phase Angle**	26	6.3 (0.9)	6.8 (1.0)†
**Total Cholesterol (mg/dl)**	30	163 (26)	142 (25)‡
**LDL Cholesterol (mg/dl)**	29	96 (17)	79 (17)‡
**HDL Cholesterol (mg/dl**	30	36 (8)	42 (9)‡
**Triglycerides (mg/dl)**	30	172 (90)	106 (47)‡

Statistically significant changes between weeks 0 to 12 and weeks 0 to 24 using Bonferroni correction with

* p<0.025,

† p<0.01, and

‡ p<0.001.
